# Monocyte/Macrophage Lineage Cells From Fetal Erythromyeloid Progenitors Orchestrate Bone Remodeling and Repair

**DOI:** 10.3389/fcell.2021.622035

**Published:** 2021-02-04

**Authors:** Yasuhito Yahara, Xinyi Ma, Liam Gracia, Benjamin A. Alman

**Affiliations:** ^1^Department of Orthopaedic Surgery, Duke University School of Medicine, Durham, NC, United States; ^2^Department of Orthopaedic Surgery, Faculty of Medicine, University of Toyama, Toyama, Japan; ^3^Department of Molecular and Medical Pharmacology, Faculty of Medicine, University of Toyama, Toyama, Japan; ^4^Department of Cell Biology, Duke University School of Medicine, Durham, NC, United States

**Keywords:** macrophage, osteoclast, yolk sac, erythromyeloid progenitors, fracture, remodeling

## Abstract

A third of the population sustains a bone fracture, and the pace of fracture healing slows with age. The slower pace of repair is responsible for the increased morbidity in older individuals who sustain a fracture. Bone healing progresses through overlapping phases, initiated by cells of the monocyte/macrophage lineage. The repair process ends with remodeling. This last phase is controlled by osteoclasts, which are bone-specific multinucleated cells also of the monocyte/macrophage lineage. The slower rate of healing in aging can be rejuvenated by macrophages from young animals, and secreted proteins from macrophage regulate undifferentiated mesenchymal cells to become bone-forming osteoblasts. Macrophages can derive from fetal erythromyeloid progenitors or from adult hematopoietic progenitors. Recent studies show that fetal erythromyeloid progenitors are responsible for the osteoclasts that form the space in bone for hematopoiesis and the fetal osteoclast precursors reside in the spleen postnatally, traveling through the blood to participate in fracture repair. Differences in secreted proteins between macrophages from old and young animals regulate the efficiency of osteoblast differentiation from undifferentiated mesenchymal precursor cells. Interestingly, during the remodeling phase osteoclasts can form from the fusion between monocyte/macrophage lineage cells from the fetal and postnatal precursor populations. Data from single cell RNA sequencing identifies specific markers for populations derived from the different precursor populations, a finding that can be used in future studies. Here, we review the diversity of macrophages and osteoclasts, and discuss recent finding about their developmental origin and functions, which provides novel insights into their roles in bone homeostasis and repair.

## Fracture Repair

A third of all individuals will fracture a bone. As we age there is an increased chance of sustaining a fracture, and the pace of repair slows. This increases the chance that a fracture goes on to a delayed or non-union. The risk of a non-union increases with age approaching 5% in some fracture types in patients over 60. Non-union is associated with decreased mobility, and this results in significant morbidity and even mortality in older individuals (Nicoll, 1964; Sarmiento et al., 1989; Kyro et al., 1993; van Staa et al., 2001; Meyer et al., 2003; Lu et al., 2005; Ekholm et al., 2006; Gruber et al., 2006; Kanakaris and Giannoudis, 2007).

Fractures heal by either primary or secondary mechanisms. Primary healing is less common, occurring when a fracture is rigidly fixed through certain types of surgery, characterized by new cortical bone laid down without any intermediate. In the more common secondary healing, immature and disorganized bone forms between the fragments, which is termed the callus (McKibbin, 1978; Perren, 1979; Arnold, 1987; Einhorn, 1998). Secondary healing progresses through three phases. In the initial phase, bleeding from the damaged tissues causes a hematoma at the fracture site, and hematopoietic cells such as macrophages reach the fracture site (Ozaki et al., 2000). The blood supply is temporarily disrupted for a few millimeters of the bone on either side of the fracture, producing local necrosis and hypoxia. This process results in the release of proteins that promote differentiation of mesenchymal cells into bone-forming cells (Onishi et al., 1998; Schmitt et al., 1999; Champagne et al., 2002; Cho et al., 2002; Kloen et al., 2002). In the proliferative phase, undifferentiated mesenchymal cells aggregate at the site of injury, proliferate, and differentiate (Arnold, 1987). This process involves both intramembranous and endochondral ossification. Intramembranous ossification involves the formation of bone directly from undifferentiated mesenchymal cells (Rozalia et al., 2005). During endochondral ossification, mesenchymal cells differentiate into chondrocytes, producing a cartilaginous matrix, which is then replaced by bone. In the last phase, extensive remodeling occurs, until the damaged skeletal element regains its original shape (McKibbin, 1978; Einhorn, 1998; Rozalia et al., 2005).

When fracture healing is impaired, osteoblast differentiation is inhibited, and undifferentiated mesenchymal tissue remains at the fracture site. This results in a delayed union, or non-union. A variety of factors, including motion at the fracture site and the age of the patient impair healing. Non-union is less common in younger individuals (Girgis and Pritchard, 1958; DeAngelis, 1975; Kwong and Harris, 2008). The pace of fracture repair slows after skeletal maturity, with 3 month old juvenile mice (equivalent to a older teenager or young adult) healing almost twice as fast as 20-month old mice (equivalent to a mid 60 year old) (Tonna, 1964; Meyer et al., 2006). *In-vitro* studies show that a smaller proportion of undifferentiated mesenchymal cells differentiate to osteoblasts in older animals, and this block to differentiation delays fracture healing (Meyer et al., 2001; Calori et al., 2007; Strube et al., 2008; Clement et al., 2011).

## Macrophage and Monocyte Cells

Macrophages were initially defined in the Early nineteenth century by Metchnikoff, a finding that contributed to his Nobel prize with Paul Ehrlich (Teti et al., 2016). These heterogenous myeloid derivatives participate in nearly every biological role from development, injury/repair processes, and homeostasis. Since their discovery, macrophages have been found to localize and inhabit many locations throughout the body (Hume and Gordon, 1983; Hume et al., 1984; Tidball and Villalta, 2010; Libby et al., 2013, 2014; Odegaard and Chawla, 2013; Ma et al., 2018). In adult mammalian organisms, bone marrow progenitor cells influenced by macrophage colony stimulating factor (M-CSF) can differentiate into monocytes and enter circulation, later entering tissue as macrophages (Akashi et al., 2000; Hettinger et al., 2013). Functionally, macrophages specialize in sentinel like functions; phagocytosing cell debris, actively promoting tissue growth, and interact closely with dendritic cells for antigen presentation (Italiani and Boraschi, 2014). However, their plasticity and variable gene expression has made these cells types difficult to study. Long term sustainability of macrophage populations is suggested to be as a result of myeloid cells, and while not yet known, potentially early embryonic precursor (Kaur et al., 2018; Yahara et al., 2020). This review will cover how these components contributes to repair, regeneration, and bone homeostasis.

There is heterogeneity in monocyte population in peripheral blood (Passlick et al., 1989). The Nomenclature Committee of the International Union of Immunologic Societies defined three major human monocyte populations (Ziegler-Heitbrock et al., 2010). The major population (~90% of blood circulating monocytes) is referred to as “Classical monocytes,” expressing high levels of cluster of differentiation 14 (CD14). Intermediate monocytes are approximately 10% of this population expressing high levels of both CD14 and CD16. A “non-classical” subset is classified by high CD16 expression and lower CD14 expression. In mice, classical monocytes are featured by the surface marker combination lymphocyte antigen 6 complex (Ly6C)^high^ CX3C chemokine receptor 1 (CX3CR1)^int^ C-C Motif Chemokine Receptor 2 (CCR2)^+^CD62L^+^CD43^low^, while “non-classical” monocytes are distinguished by the Ly6C^low^CX3CR1^high^CCR2^low^CD62L^−^CD43^+^. Classical monocytes have a lifespan of about 1 day, while non-classical monocytes live about 2 days in mice and 7 days in humans (Yona et al., 2013; Patel et al., 2017).

Ly6C^high^CX3CR1^int^ classical monocytes, previously called inflammatory monocytes, are a transient population of cells with a wide variety of differentiation potential. Classical monocytes shift into the circulation from the bone marrow during the steady-state to replenish the tissue-resident macrophages. However, the epidermis (Chorro et al., 2009), the central nervous system (Ajami et al., 2007; Mildner et al., 2007; Ginhoux et al., 2010), and the alveolar space (Guilliams et al., 2013; Hashimoto et al., 2013) are characterized by the limited or no monocyte engraftment, because of the high self-renewal potential of the local tissue-resident macrophages or the restricted blood access to their locations. Ly6C^low^CX3CR1^high^ non-classical monocytes, previously called patrolling monocytes, can adhere and crawl along capillary endothelial cells to monitor vascular integrity (Auffray et al., 2007). In the steady-state, Ly6C^high^CX3CR1^int^ classical monocytes are able to re-enter the bone marrow and convert to Ly6C^low^CX3CR1^high^ cells (Varol et al., 2009).

## Role of Macrophages in Fracture Repair

Macrophage responsiveness to external environmental stimuli is essential for keeping pathogens at bay and ameliorating damaged tissue (Shapouri-Moghaddam et al., 2018). While macrophages had been described existing as either classical (inflammatory response) or alternative activation (resolving response), cumulative evidence has points toward macrophages inhabiting a spectrum of plasticity between the two polar extremes of “M1” and “M2”. Classically activated macrophages (M1) are stimulated through a variety of processes. Bacterial or viral infections may initiate cell-cell mediated responses, which help to produce activating molecules like interferon, lipopolysaccharide, or toll-like receptor. Early studies found that M1 like macrophages are able to enhance woven bone formation through MSC (mesenchymal stromal cell) interactions. Alternatively, activated macrophages (M2) assist in anti-inflammatory signals and help promote processes like angiogenesis and revascularization of new mineral deposits. These cells are abundant during bone healing processes (Biswas et al., 2012; Murray et al., 2014; Sivaraj and Adams, 2016; Murray, 2017; Atri et al., 2018; Nathan et al., 2019).

Macrophages in bone are referred to as osteomacs. During development, these macrophages associate closely with osteoblasts during new tissue formation (Winkler et al., 2010). After an injury, macrophages play roles orchestrating an immune response, causing inflammation, and eventually contributing resolution factors that finalize wound healing (Pajarinen et al., 2019). This has been observed in many organs (Naito, 1993; T'Jonck et al., 2018; Xie et al., 2019). During bone fracture, an immune response is rapidly activated with macrophages being one of the first cells at the fracture site (Mosser and Edwards, 2008). These macrophages are activated toward an M1 phenotype recruiting other effector cells to the site of injury. Macrophages also work to clear debris as revascularization tissue forms creating granulated tissue (Thomas and Puleo, 2011; Baht et al., 2018). After the initial phase of inflammation, macrophages begin to help form a soft cartilaginous callus. After depleting macrophages at the time of femoral fracture, there is a complete lack of callus formation. Similarly, depletion during the anabolic stages of repair also showed impaired callus formation and an inability to properly restore bone integrity (Raggatt et al., 2014). Studies by Vi et al. expanded the understanding of macrophage-bone communication during osteoblast differentiation. By depleting macrophage cells during development, it was found that animals had a substantially reduced bone mineral density and maintenance of mesenchymal progenitors was compromised (Vi et al., 2015).

In intramembranous repair, macrophages have been identified to closely associate with the fracture site throughout regeneration (Sinder et al., 2015). Similar to endochondral bone formation, macrophage fas-induced apoptosis (MAFIA) mice deficient in macrophages had bone which lost healing robustness (Alexander et al., 2011). A recent study using both MAFIA mice and clodronate liposome depletion models saw a loss in woven bone integrity. Alexander et al. had also shown that macrophages serve a temporal role throughout bone repair. Evidence had suggested that macrophage depletion at the time of injury resulted in a worse phenotype compared to earlier time points (Lin and O'Connor, 2017).

Macrophage function changes with age. A blunted response to stimuli and general hyperinflammation is often observed with aging (Strube et al., 2008; Tarantino et al., 2011). Early studies showed that the polarization of macrophages and their plasticity is blunted with age (Loi et al., 2016). Data from the study of old rats show that M2 macrophages lose their anti-inflammatory abilities (Löffler et al., 2019). Examination of *Ccr2* deficient mice show that the angiogenic capacity of M2 macrophages is important in the remodeling process (Xing et al., 2010). This angiogenic capacity is associated with the ability to dissociate collagen matrices (Moldovan et al., 2000). The pace of fracture healing slows with age. Using heterochronic parabiosis (Vi et al., 2018), it was shown that macrophages from young mice showed rejuvenating effects on fracture repair when circulated into old mice. Furthermore, macrophage cells from old mice slowed the pace of repair in young animals.

It has been thought that stimulating M2 macrophages while inhibiting M1 polarization will improve regeneration. However, macrophages recruited sites of injury exhibit a plastic state and can self-modulate toward M2 phenotypes. Indeed, as reviewed by Pajarinen et al. M1, macrophages can serve both a beneficial role and inhibitory role during regeneration (Pajarinen et al., 2019), supporting the notion that the specific M1 or M2 state regulates regenerative capacity is an oversimplification.

## Ontogeny of Macrophages

Macrophages have several embryonic origins. They can derive from yolk sac (YS) posterior plate mesoderm directly, an erythro-myeloid progenitor (EMP) that derives from the YS hemogenic endothelium, or from a hematopoietic stem cell. Their embryonic development occurs in what is often termed “waves” (McGrath et al., 2015a; Munro and Hughes, 2017). Their embryonic development occurs in what is often termed “waves” (McGrath et al., 2015a; Munro and Hughes, 2017). In mice, primitive hematopoiesis starts as a first wave around embryonic day 7 (E7) in the blood island of the YS and directly seed embryonic tissues (Moore and Metcalf, 1970; Palis et al., 1999; Ginhoux and Guilliams, 2016; Hoeffel and Ginhoux, 2018; Lee et al., 2018). The YS hemogenic endothelium gives rise to EMPs that can differentiate into fetal macrophages. EMPs appear around E7-7.5 in the yolk-sac (Ginhoux et al., 2010; Italiani and Boraschi, 2017) and can differentiate into colony stimulating factor 1 receptor (CSF1R)^+^ yolk-sac macrophages at E8.5 (Gomez Perdiguero et al., 2015; Hoeffel and Ginhoux, 2018). While EMPs were initially described to arise from the yolk sac endothelium, just prior to vascular remodeling, they can also emerge directly from endothelial cells. Early c-Myb-independent EMPs that give rise to primitive yolk sac macrophages without passing through monocyte intermediates and a late c-Myb-dependent EMPs that seed the fetal liver to produce fetal monocytes (Hoeffel et al., 2015). This first-wave, in which EMPs/primitive myeloid precursors development occurs independent of the transcriptional activator c-Myb, is instead dependent on PU.1 (Schulz et al., 2012; Gomez Perdiguero and Geissmann, 2013; Ginhoux and Guilliams, 2016). Myb-independent EMPs can differentiate into CX3CR1 positive YS macrophages at E8.5, also called premacrophage (pMac), resulting in a source of tissue-resident macrophages (Mass et al., 2016). Mouse EMPs are generated from Tie2+ yolk sac ancestors when the heart tube begins to contract just prior to vascular remodeling (Frame et al., 2013; Gomez Perdiguero et al., 2015). EMPs are also present in other hemogenic tissues, such as the placenta and umbilical cord, but at a much lower frequency than the yolk sac (Dzierzak and Speck, 2008). Refinement of markers will help better define the various progenitor calls and how they can differentiate For instance, Expression of kit, aa4.1, cd41, cd45 may mark lympho-myeloid progenitors in the yolk sac (Yamane et al., 2009).

A second wave of EMPs develop from E8.25 adopt monocyte stage in the fetal liver. At E9, EMPs that differentiate through this process a Runx1-dependent endothelial-to-hematopoietic transition (Yzaguirre et al., 2017). Endothelial EMPs elongate and integrate in the endothelium and further asymmetrically divide. One of the daughter cells remains in the vessel wall, whereas the other enters circulation. During this process, blood flow facilitates the transition of EMPs from the endothelium into circulation through a nitric oxide-dependent mechanism but is not required for differentiation (Kasaai et al., 2017). EMPs migrate to the fetal liver where they can differentiate into multiple hematopoietic cell types including fetal liver monocytes (Hoeffel et al., 2015). At E10.5, EMPs are found in the fetal liver, which indicates that EMPs migrate to the fetal liver after entering circulation and starts to rapid differentiation here (McGrath et al., 2015b).

The fetal liver niche provides critical cues or an environment for CSF1R+ EMPs to give rise to pMacs (pre-macrophage cells). Although previous studies indicated that only adult HSCs are dependent on c-Myb expression, recent studies show that late EMP in the fetal liver requires c-Myb to contribute to the fetal monocytes (Hoeffel et al., 2015; Hoeffel and Ginhoux, 2018). Fate-mapping analysis of EMP differentiation indicated that pMacs lose Kit expression and increase CD45 expression (Gomez Perdiguero et al., 2015). Although the location where CSF1R+ EMPs acquire CX3CR1 expression is not clear, upregulated CX3CR1 is found in fetal liver pMacs. CX3CR1^+^ pMacs then rapidly proliferate and gain access to the bloodstream to migrate toward the embryo. Colonization of the head, caudal region, and limbs is delayed in CX3CR1-deficient embryos, indicating tissue colonization by pMacs is dependent on CX3CR1 (Mass et al., 2016). Intravital microscopy revealed that trafficking of EMPs and pMacs from the yolk sac to liver primordium and other organ rudiments peaks around E10.5, dramatically decreases toward E12.5 and is no longer evident from E14.5 onwards (Stremmel et al., 2018). As pMacs invade the embryo organ rudiments, Elvira Mass et al. revealed a core macrophage transcriptional program by scRNA-seq and bulk RNA-seq that genes differentially expressed during differentiation from pMacs to fetal and postnatal organ-specific tissue-resident macrophage before acquiring F4/80 expression (Mass et al., 2016). In the third wave, hematopoietic stem cell precursors (pro-HSCs) emerge in the aortogonado-mesonephros region at E10.5 and differentiate to embryonic HSCs at E12.5, which shift later to the bone marrow (Ginhoux and Guilliams, 2016). Bone marrow HSCs eventually establish the circulating monocyte-derived macrophages (Italiani and Boraschi, 2017). The adult bone marrow HSCs-derived monocyte and macrophages are fundamentally different cell populations from embryonically established tissue-resident macrophages.

While it was initially thought that tissue-resident macrophage populations are replenished by monocytes from the blood, more recent lineage tracing data shows that many adult resident macrophages are instead established during development and maintain themselves in the postnatal tissue by proliferation (Epelman et al., 2014; Gomez Perdiguero et al., 2015). EMPs are the first definitive hematopoietic stem cell (HSC)-independent cells and are the source of these adult/postnatal tissue-resident macrophages.

In human embryos, the yolk sac serves as the initial site of hematopoiesis and gives rise to macrophage progenitors. Early studies found that human myeloid precursors emerge in the human yolk sac and migrate to the fetal liver during 28–35 days post-conception (Migliaccio et al., 1986). Given that at this time neither the human fetal liver possess repopulating ability nor HSC start colonization, the cells seeding the early fetal liver are likely to include yolk sac-derived EMPs (Ivanovs et al., 2017). Recent studies confirmed the expression of CX3CR1 in human yolk sac-derived pMacs as in mice (Stremmel et al., 2018). Although the yolk sac EMPs wave has not been formally characterized during human development, the analogies discovered between mice and human hint the conservation in their process of EMP differentiation into tissue-resident macrophage. However, previous studies uncovered that epidermal growth factor module-containing mucin-like receptor 2 (EMR2, CD312) is upregulated during differentiation and maturation of human macrophages, while mice lacks *Emr2* gene (Kwakkenbos et al., 2006; Chang et al., 2007). Thus, the differences between mice and human EMP differentiation into tissue-resident macrophage may exist and are areas for future exploration.

## Osteoclasts and Hematopoietic Stem cell Differentiation

Osteoclasts are derived from the monocyte/macrophage lineage and are responsible for the resorption of bone tissues (Udagawa et al., 1990; Takahashi et al., 1994). They undergo cell-cell fusion to form multinucleated cells under the influence of the receptor activator of the nuclear factor-κB ligand (RANKL) (Lacey et al., 1998; Yasuda et al., 1998). Osteoclasts can originate from the HSCs in the bone marrow. HSCs have the capacity to self-renew and differentiate into each hematopoietic cell type (Spangrude et al., 1988). While the traditional concept of linear development of HSCs down a “hematopoietic differentiation tree” has been being challenged by recent studies, this general framework is useful in understanding how macrophage cells and osteoclasts develop from HSCs(Laurenti and Gottgens, 2018). Self-renewing HSCs give rise to multipotent progenitors (MPPs) that in turn generate the lineage-restricted precursors (Kawamoto et al., 2010; Seita and Weissman, 2010). The precursors then bifurcate into oligopotent progenitors, common myeloid progenitors (CMPs), and common lymphoid progenitors (CLPs). The CMPs develop into megakaryocyte/erythrocyte progenitors, and the granulocyte (GR)/macrophage progenitors (GMPs). Further, the GMPs differentiate into a common macrophage/osteoclast/dendritic cell progenitor (MODP) that later produces osteoclasts under the influence of RANKL and colony-stimulating factor-1 (CSF-1) (Arai et al., 1999; Miyamoto et al., 2001). However, emerging single-cell transcriptome technologies, and studies of stem cell differentiation, are challenging the traditional view of the hematopoietic hierarchy. These studies have found additional levels of plasticity, and unipotent stem cells (Notta et al., 2016; Laurenti and Gottgens, 2018; Jacobsen and Nerlov, 2019). As additional studies confirm and build on these findings, our view of HSC differentiation will be refined.

The hematopoietic transcription factor, PU.1 (encoded by the *Spi-1* gene), regulates the initial step of myeloid differentiation; it also regulates the CSF1R and receptor activator of nuclear factor-κ B (RANK) gene expression in myeloid progenitors (Tondravi et al., 1997; Kwon et al., 2005; Ishiyama et al., 2015). Followed by the subsequent activation of RANK on the surface of osteoclast precursors, the RANKL-RANK signaling recruits tumor necrosis factor receptor-associated factor 6 (TRAF-6) to modulate a variety of signaling cascades (Kobayashi et al., 2001), such as the canonical and non-canonical nuclear factor-kappa B (NF-kB) pathway, calcium signaling (Sato et al., 2006), and mitogen-activated protein kinase (MAPK) pathway that includes protein kinases, such as extracellular signal-regulated kinase (Miyazaki et al., 2000), Janus N-terminal kinase, p38, and phosphatidylinositol 3-kinase PI3K. This leads to the activation of many transcription factors related to the osteoclast formation, including activator protein 1 (Matsuo et al., 2000) and nuclear factor of activated T cell cytoplasmic 1 (NFATc1) (Takayanagi et al., 2002). Cell surface receptors, such as triggering receptor expressed in myeloid cells-2 (TREM2) and osteoclast-associated receptor also transmit intracellular signals and trigger the phosphorylation of spleen tyrosine kinase, resulting in the Ca2+ mobilization and activation of NFATc1 (Koga et al., 2004). Thus, these factors stimulate the transcriptional activation of osteoclast-specific genes, such as tartrate resistant acid phosphate (TRAP, encoded by the *Acp5* gene), cathepsin K, and matrix metalloproteinase-9, and coordinate the differentiation and maturation of mononuclear osteoclast precursors into bone-resorbing mature osteoclasts.

## Osteoclasts From Embryonic Myeloid Progenitors

**Figure 1:**
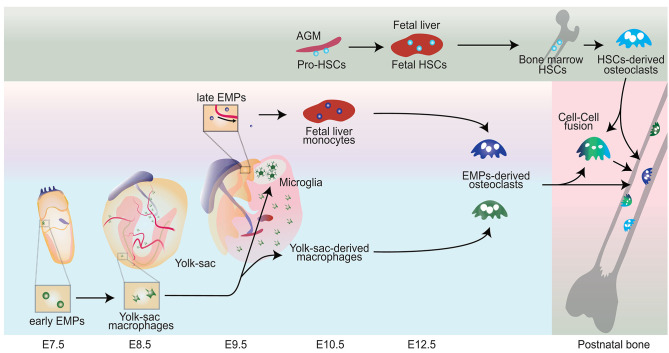
Schematic representation showing the origin of osteoclasts. Early erythromyeloid progenitors (EMPs) appear around E7-7.5 in the yolk-sac and differentiate into yolk sac macrophages without passing through monocyte intermediates. CX3C chemokine receptor 1 (CX3CR1) positive pre-macrophages produce a significant source of yolk-sac-derived macrophages. Late EMPs emerge in the yolk-sac at E9 and migrate to the fetal liver to produce fetal liver monocyte. Hematopoietic stem cell precursors (pro-HSCs) emerge at E10.5 and migrate to the fetal liver around E12 and turns to fetal HSCs, which later shift to the bone marrow. Bone marrow HSCs eventually can establish HSCs-derived osteoclasts. Fetal liver monocytes and yolk sac derived macrophages can differentiate into osteoclast (EMPs-derived osteoclasts) in the neonatal bone with possible cell-cell fusion with HSCs-derived Osteoclasts.

Osteoclasts were initially thought to derive from the circulating monocyte lineage progenitor cells in the bone marrow. However, recent lineage tracing studies show that osteoclast precursors arise from the yolk sac (Yahara et al., 2020) questioned the osteoclast ontogeny (Gomez Perdiguero et al., 2015; Mass et al., 2016) (Figure 1). Jacome-Galarza et al. found that the mice lacking RANK or CSF-1R in both EMPs and HSCs lineage developed severe bone disease and failure of tooth eruption at a young age. However, the mice lacking RANK or CSF-1R in only HSCs lineage showed normal eruption of tooth and no phenotypic defect in the bone at a young age (4 weeks), but increased bone mass after 16 weeks of age. These results suggest that the EMP-derived osteoclasts are essential for bone development and tooth eruption, and they are gradually replaced by the HSC-derived progenitor cells (Jacome-Galarza et al., 2019). We recently reported that macrophages originating from the EMPs in the yolk sac produce neonatal osteoclasts that can provide a space for the postnatal bone marrow and gives rise to a population of long-lived osteoclast precursors. This population contributes to bone remodeling at steady state and fracture healing. Furthermore, the data from cell-fate analyses of EMP and HSC lineages indicated the possibility of cell-cell fusion between these two lineages. The yolk sac-derived macrophages can migrate through the bloodstream to bone after an injury (Yahara et al., 2020). These osteoclasts are also involved in the bone remodeling of other pathologic conditions, such as rheumatoid arthritis and metastatic bone disease.

## Chemoattraction of Myeloid Cells During Fracture Repair

Proteins secreted during early inflammation are critical for the successful healing of bone fractures, as they initiate a cascade leading to skeletal repair. Chemoattractants, such as chemokines and cytokines, attract inflammatory cells to the site of tissue damage. Neutrophils are the primary cells that arrive at the site of injury, in response to the localized secretion of damage-associated molecular patterns (DAMPs) (Zhang et al., 2010) that facilitate the release of hydrogen peroxide (H2O2) and guide the further recruitment of neutrophils. DAMPs, including the high-mobility group box 1 (HMGB1) proteins, N-formyl peptides, S100 proteins, and heat shock proteins, are mainly recognized by the nucleotide oligomerization domain (NOD)-like receptors (NLRs) and toll-like receptors (TLRs) (Vourc'h et al., 2018; Relja and Land, 2020). Further, the neutrophils and other immune cells induce monocyte chemotaxis via the secretion of several CXC chemokine ligands (CXCL1, CXCL2, CXCL3, CXCL8, CXCL10, and CXCL12) (Kitaori et al., 2009; Kolar et al., 2011; Myers et al., 2015; Förster et al., 2016; Hoff et al., 2016; Furman et al., 2018; Burska et al., 2020) and CC chemokine ligands (CCL2, CCL3, CCL4, and CCL5)(Xing et al., 2010; Wu et al., 2013; Ishikawa et al., 2014; Hoff et al., 2016) during fracture healing.

CCL2, also known as monocyte chemotactic protein-1 (MCP-1), and its receptor CCR2, are involved in the recruitment of neutrophils (Johnston et al., 1999), monocytes/macrophages (Tsou et al., 2007; Xing et al., 2010; Arakaki et al., 2016; Biguetti et al., 2018), and mesenchymal progenitor cells (Ishikawa et al., 2014) during inflammation and fracture repair. Classical Ly6C^high^ monocytes, also called as pro-inflammatory monocytes, represent about 2–5% of the circulating white blood cells in a steady state and express high levels of CCR2, but low levels of CX3CR1 (Geissmann et al., 2003). Once inflammation or injury occurs, the Ly6C^high^ and CCR2^high^ monocytes rapidly infiltrate the site of injury via CCR2-CCL2 signaling that attracts other inflammatory monocytes from the bone marrow or blood circulation to the site and promotes their differentiation to macrophages. It has been reported in previous studies that the expression level of CCR2 and its ligand, CCL2, significantly increased during the acute phase of fracture healing (Xing et al., 2010; Ishikawa et al., 2014). In addition, mice lacking the *CCR2* gene showed impaired recruitment of monocytes and macrophages to the site of injury, as well as delay in callus remodeling, cartilage maturation, and endochondral ossification (Xing et al., 2010). Thus, the impaired recruitment of macrophage in the initial step of fracture healing may be able to affect the attraction and differentiation of osteo-chondro progenitors, resulting in the delayed fracture healing. Both CCR2 and CCL2-null mice exhibited an increase in bone mass due to insufficient osteoclast formation and bone resorption (Binder et al., 2009; Sul et al., 2012). However, there was no significant difference in the number of osteoclasts at the fracture callus in *CCR2*^−/−^ mice as compared to that in the wild type mice (Xing et al., 2010). The possible explanations of this phenomenon could be that other molecules may be involved in orchestrating the attraction of osteoclast precursors to the site of fracture during bone healing, or the osteoclasts that participate in the fracture callus remodeling may not be the same population as found during physiological conditions. The molecular mechanisms important for macrophage cell migration and how they are triggered by chemoattractant is an area in which continued investigation is needed.

## Role of Osteoclasts in Callus Remodeling

Secondary fracture repair progresses through endochondral ossification (Gerstenfeld et al., 2003; Zhang et al., 2016). Chondrocytes derived from the skeletal stem cells or mesenchymal progenitors (Nakahara et al., 1990; Chan et al., 2013, 2015, 2018; Worthley et al., 2015; Yue et al., 2016; Mizuhashi et al., 2018) differentiate into hypertrophic chondrocytes and mineralize the cartilage matrix, resulting in the initial soft cartilaginous callus. While it was previously thought that hypertrophic chondrocytes undergo apoptosis, recent studies using the genetic lineage tracing mice model revealed that the chondrocytes could directly differentiate into osteoblast lineage cells during both the physiological growth phase and fracture repair (Ono et al., 2014; Yang et al., 2014a,b; Zhou et al., 2014). Bone regeneration is orchestrated by the invading-osteoblasts along with vasculature that is derived from the mesenchymal progenitors of the periosteum (Nakahara et al., 1990; Murao et al., 2013; Duchamp de Lageneste et al., 2018), bone marrow mesenchymal stromal cells (Sekiya et al., 2002), and/or the cells from terminally differentiated chondrocytes (Zhou et al., 2014; Hu et al., 2017). Soft callus remodeling is a process of gradual removal of the cartilage/fibrocartilage and its systematic replacement with woven bone. The woven bone is subsequently transformed into a lamellar bone, also called as a hard callus, in the final step of fracture healing.

Several animal models were used to examine the role of osteoclasts during callus remodeling (Table 1). The disruption of osteoclastogenesis by pharmacological substances, such as RANKL inhibitors (RANK-Fc and denosumab), osteoprotegerin (OPG), bisphosphonates (alendronate and zoledronic acid), clodronate liposomes, and cathepsin K (CTSK) inhibitors, differentially affected the callus remodeling (Flick et al., 2003; Ulrich-Vinther and Andreassen, 2005; Gerstenfeld et al., 2009; Soung do et al., 2013; Pennypacker et al., 2016; Lin and O'Connor, 2017). Treatment with alendronate and zoledronic acid did not reduce the number of osteoclasts in the callus and serum tartrate-resistant acid phosphatase (TRACP) 5b level, but increased its volume and the cartilaginous callus, indicating delayed callus remodeling (Gerstenfeld et al., 2009; Soung do et al., 2013). Similarly, the treatment with RANK-Fc, denosumab, and OPG delayed the cartilage resorption and remodeling due to impaired differentiation of osteoclasts in the fracture callus (Flick et al., 2003; Ulrich-Vinther and Andreassen, 2005; Gerstenfeld et al., 2009). Both alendronate and denosumab retarded the following: fracture callus remodeling, elimination of cartilage (Soung do et al., 2013), improvement in mechanical strength, and bone mineral content (BMC), as compared to those in the control groups, during fracture repair (Gerstenfeld et al., 2009). Although the pharmacological inhibition of osteoclastogenesis delays the callus remodeling and forms a large woven bone callus, it may help to provide mechanical support at the fracture site, rather than a remodeled lamellar callus. Genetic ablation of RANK and CSF1 showed osteopetrotic (op) phenotype and affected the fracture callus remodeling and healing. The RANK knockout (KO) and op/op mice lacked osteoclasts but showed radiographic and histological evidence of callus formation (Flick et al., 2003). This data indicated that osteoclasts are not essential for fracture callus formation. However, the bone healing rate was reduced in the RANK KO mice as compared to that in the osteopetrotic op/op mice. The OPG KO mice developed an increased number of osteoclasts in the fracture callus. The accelerated resorption of cartilaginous callus in OPG KO mice promoted fracture healing (Ota et al., 2009). In addition, the pharmacological and genetic ablation of osteoclast progenitor cells (macrophage and monocyte) also delayed fracture healing. The clodronate liposome treatment did not prevent the callus formation, but it reduced the number of osteoclasts and delayed the callus cartilage remodeling (Lin and O'Connor, 2017). Ablation of the lysozyme-M-positive cells using LyzM-Cre in diphtheria toxin subunit A-expressing (DTA) mice suppressed both cartilage and bony callus formation while accelerating fibrosis, thus, resulting in delayed fracture healing (Vi et al., 2015). Interestingly, constitutive macrophage deficiency did not affect the number of TRAP+ osteoclasts in the fracture callus. The possible reason for this observation is that osteoclasts of different sources may have contributed to the callus remodeling during fracture repair. Pharmacological inactivation or genetic ablation of CTSK led to an increase in the number of osteoclasts in the fracture callus, high bone formation and strength, and an increase in the bone mineral density (Ota et al., 2009; Soung do et al., 2013; Gentile, 2014; Pennypacker et al., 2016). CTSK inhibitors have a potential to inhibit the bone resorption ability of osteoclasts without changing the ability of bone synthesis (Soung do et al., 2013; Pennypacker et al., 2016). Therefore, odanacatib (ODN), which is a selective oral inhibitor of CTSK, has been considered as a strong candidate for the treatment of osteoporosis and a potent inhibitor of osteoclastic activity. Although ODN diminished the risk of fractures, it was related to elevated cardiovascular risks, such as strokes, especially in osteoporotic postmenopausal women. Thus, on analyzing the overall benefits and risks associated with the ODN drug, the investigators of the study decided to discontinue the use of ODN for the treatment of osteoporosis (McClung et al., 2019).

**Table 1:** Animal models to examine the disruption of osteoclastogenesis during fracture healing.

***Pharmacological agents***	**Species**	**Age**	**Sex**	**Locus**	**Method**	**Post-operative fixation**	**OC number**	**Callus formation**	**BMD or BMC**	**Mechanical testing**	**Bone healing**	**References**
Alendronate	Mouse	8-17 W	M	Femur	Three-point bending	Intramedullary pin	↑	↑	↑	↑	N/A	Gerstenfeld et al., 2009
Denosumab	Mouse	8-17 W	M	Femur	Three-point bending	Intramedullary pin	↓	↑	↑	↑	N/A	Gerstenfeld et al., 2009
Osteoprotegerin (OPG)	Rat	3 M	F	Tibia	Three-point bending	Intramedullary pin	↓	→	↑	→	N/A	Ulrich-Vinther and Andreassen, 2005
RANK:Fc (high doze)	Mouse	12 W	N/A	Tibia	Three-point bending	Intramedullary pin	↓	↑	N/A	→	→	Flick et al., 2003
Clodronate liposome	Mouse	10-12 W	F	Femur	Three-point bending	Intramedullary pin	↓	↑	N/A	→	↓	Lin and O'Connor, 2017
CTSK-I (L-235)	Mouse	7-8 W	M	Femur	Three-point bending	Intramedullary pin	↑	↑	↑	N/A	N/A	Soung do et al., 2013
Alendronate	Mouse	7-8 W	M	Femur	Three-point bending	Intramedullary pin	→	↑	↑	N/A	N/A	Soung do et al., 2013
Odanacatib	Rabbit	9 M	F	Ulnar	Low-speed bone saw	Splint	↑	→	↑	↑	↑	Pennypacker et al., 2016
***Genetic mouse model***												
RANK KO	Mouse	12 W	N/A	Tibia	Cutting with scissors	Unstabilized	↓	→	N/A	N/A	↓	Flick et al., 2003
Op/Op	Mouse	12 W	N/A	Tibia	Cutting with scissors	Unstabilized	↓	N/A	N/A	N/A	→	Flick et al., 2003
Lyz-Cre; DTA	Mouse	12 W	N/A	Tibia	Cutting with scissors	Intramedullary pin	→	↓	N/A	N/A	↓	Vi et al., 2015
CTSK KO	Mouse	8-10 W	F	Femur	Three-point bending	Intramedullary pin	↑	→	↑	↑	↑	Gentile, 2014

*RANK, receptor activator of nuclear factor-κB; CTSK-I, cathepsin K-inhibitor; KO,knockout; op/op, colony-stimulating factor 1(CSF-1)-less osteopetrotic mouse; W, week-old; M, male; F, female; OC, osteoclast; BMD, bone mineral density; BMC, bone mineral content; N/A, not applicable*.

Traditionally, hard bony callus remodeling has been thought to be the final step in fracture repair. However, Takeyama et al. using the medaka fin ray fracture model, recently demonstrated that two types of functional osteoclasts are activated in the different phases of fracture healing (Takeyama et al., 2014). Immediately after a fracture, the early-induced osteoclasts, which are of small size and have high morbidity with low TRAP activity, localize on the edge of the bone fragments. However, the late-induced osteoclasts start to appear at the inner surface of the callus with high TRAP-activity and large morphology. We found that fms-like tyrosine kinase 3 (FLT3)-positive progenitors of the HSCs lineage could migrate to the injury site and differentiate into TRAP+ and Vpp3+ osteoclasts, thereby contributing to the early phase bone resorption during bone repair in mice. The CX3CR1^+^ EMP-derived osteoclast precursors can migrate to the site of injury through blood circulation and differentiate into multinucleated osteoclasts that take part in the later phase of callus remodeling (Yahara et al., 2020). Additionally, Novak et al. showed that the circulating CX3CR1^high^ osteoclast precursor cells could migrate through blood circulation to the fracture callus and differentiate into TRAP-positive mature osteoclasts in the later phase of callus remodeling (Novak et al., 2020). In summary, each subset of osteoclasts has a distinct morphology, feature, and origin, which suggests that they have specialized and phase-specific functions.

## Mobilization of Osteoclast Precursors From Extramedullary Organs: The Spleen as a Reservoir for Embryonic Cells

Besides the bone marrow, cells from other extramedullary organs can also form mature osteoclasts *in vitro* (Boyle et al., 2003; Lianping and Edward, 2005). However, the contribution of the extramedullary reservoir to the osteoclast pool for bone homeostasis and repair has not been fully understood. Since osteoclasts as well as macrophages and monocytes have common precursor cells, several studies have shown that the extramedullary reservoir of macrophages and monocytes also plays an essential role in tissue inflammation and repair. Hoyer et al. observed that localized damage stimulated tissue macrophages in distant organs, which aided in the recovery from systemic complications after myocardial infarction, stroke, and sepsis (Hoyer et al., 2019). Wang et al. found that GATA6+ macrophages migrated directly from the peritoneal cavity in response to liver injury and contributed to the tissue repair (Wang and Kubes, 2016). Swirski et al. reported that splenic monocytes that reside in the subcapsular region of the red pulp increase their motility and accumulate at the site of ischemic myocardial injury (Swirski et al., 2009). Furthermore, Sabatel et al. reported that lung interstitial macrophages arise from the splenic monocytes by interlukin-10 (IL-10) signaling in a CCR2-independent manner during allergic airway inflammation (Sabatel et al., 2017). These data clearly showed that acute inflammation and injury caused the mobilization of monocytes and macrophage from the extramedullary organs to the site of injury.

In fracture healing, the spleen is a reservoir of osteoclast precursors. The spleen is a central lymphoid organ that has multiple functions, including the removal of cellular debris, hematopoiesis, recycling of red blood cells, and activation of the immune system during infection and inflammation. Osteoclast precursors that reside in the spleen can migrate to the bone cavity and change into mature osteoclasts (Kotani et al., 2013). Splenectomy inhibited macrophage recruitment and reduced the number of osteoclasts at the site of fracture in a rat model. Patients with fractures who received splenectomy had a significantly lower number of blood monocytes and reduced bone density than patients with fractures who did not undergo splenectomy (Xiao et al., 2017, 2018).

The spleen sustains the embryonic macrophage population derived from the EMPs in the red pulp. The red pulp macrophages are produced, at least in part, during embryonic development and are subsequently maintained throughout adulthood (Schulz et al., 2012; Hashimoto et al., 2013; Yona et al., 2013; Epelman et al., 2014). These EMP-derived macrophages can travel through the bloodstream and differentiate into osteoclasts to participate in bone remodeling during fracture repair (Yahara et al., 2020). However, the mechanism of orchestration and mobilization of EMP-derived macrophages from the spleen to the fracture site have not been elucidated. Nakamichi et al. found that IL-34 signaling induced the mobilization of osteoclast precursors from the spleen of osteopetrotic op/op mice (Nakamichi et al., 2012). Further, in a recent *in vivo* embryogenesis study of zebrafish, a CRISPR/Cas9-based reverse genetic screening also identified IL-34 as a regulator of the distribution of tissue macrophages; IL-34 can mobilize yolk sac macrophages to other embryonic tissues (Kuil et al., 2019). The contribution of circulating osteoclast precursors to *in vivo* osteoclasts pool in steady state and disease is an area of controversy. Jacome-Galarza et al. demonstrated that circulating blood monocytic cells are a major source of osteoclasts in steady condition. On the other hand, Novak et al. argued that the bone tissue is relatively protected from engraftment of circulating osteoclast precursors under steady conditions (Novak et al., 2020). There are several studies which show that the engraftment and maturation of circulating osteoclasts into mature osteoclasts is increased in fracture repair (Novak et al., 2020) and during bone resorption(Kotani et al., 2013). Thus, additional studies are needed to reveal the mechanisms that orchestrate the mobilization of circulating osteoclasts precursors from the extramedullary organs to bone in homeostasis and disease.

## Cell Fusion and Multinucleation of Osteoclasts

Osteoclasts are formed by the cell-cell fusion of mononuclear osteoclast precursors (Jansen et al., 2012). Cell-cell fusion and their multinucleation are essential for osteoclast maturation and maintenance of bone homeostasis (Li et al., 2018). The fusion of mononuclear osteoclast precursors is carried out by extra and intracellular dynamics of the interaction of various molecules. Mononuclear osteoclast precursors migrate to the bone tissue by chemotaxis through blood circulation or directly from the bone marrow. RANKL and vascular endothelial growth factor promote chemotaxis through an extracellular signal-regulated kinase 1/2-dependent pathway (Henriksen et al., 2003). Transforming growth factor β, which is released from the extracellular matrix during bone resorption, also has the potential to accelerate osteoclast recruitment through phosphatidylinositol-3 kinase (PI3K) and MAPK signaling pathways (Pilkington et al., 2001). Stromal cell derived factor-1 (SDF-1), also known as CXCL12, is critical for cell migration (Yu et al., 2003; Kollet et al., 2006; Gronthos et al., 2007). PI3K activates the transcription of SDF-1, resulting in the migration of osteoclast precursors via C-X-C chemokine receptor type 4 (CXCR4) (Adapala et al., 2019). Sphingosine-1-phosphate, a lipid mediator enriched in blood and lymph, can induce and regulate the homing of osteoclast precursors to bone (Ishii et al., 2009; Kikuta et al., 2013). Indeed, the monocyte-specific conditional sphingosine-1-phosphate knockout mice exhibited osteoporotic phenotype due to increased osteoclast activity and attachment to the bone surface (Ishii et al., 2009).

For cell-cell fusion, the migrated monocular osteoclast precursors must be in close proximity to each other and adhere (Pereira et al., 2018). The αvβ3 integrin is expressed in osteoclasts and has been implicated in cell migration as well as the formation of the sealing zone (Nakamura et al., 1999; McHugh et al., 2000). E-cadherin is a cell surface glycoprotein responsible for cell-cell adhesion and IL-4-driven macrophage fusion (Mbalaviele et al., 1995; Van den Bossche et al., 2009). During membrane fusion and multinucleation, dendritic cell-specific transmembrane protein (DC-STAMP) and osteoclast stimulatory transmembrane protein (OC-STAMP) are essential regulators of the osteoclast cell-cell fusion (Yagi et al., 2005; Miyamoto et al., 2012; Khan et al., 2013). CD44, CD47, syncytin-1, Pin1 (peptidyl-prolyl cis-trans isomerase NIMA-interacting 1), and the tetraspanins (CD9 and CD81) are also involved in osteoclast fusion and multinucleation (Sterling et al., 1998; Takeda et al., 2003; Cui et al., 2006; Søe et al., 2011; Islam et al., 2014; Møller et al., 2017). Moller et al. showed that CD47 accelerated cell fusion involving mononucleated osteoclast precursors (Møller et al., 2017). On the other hand, Synchitin-1 facilitated the fusion of multinucleated osteoclasts but regulated a reduced number of fusions between mononucleated osteoclast precursors. Lee et al. reported that mice deficient in the d2 isoform of vacuolar (H+) ATPase (v-ATPase) V0 domain (ATP6V0D2) had disrupted osteoclast fusion and increased bone formation (Lee et al., 2006). Thus, osteoclast fusion is not a random process, but there is a strict mechanism for the selection of fusion partners based on the heterogeneity of the osteoclast precursors and surrounding environmental cues.

## Cell Fusion Between EMPs- and HSCs-Derived Osteoclast Precursors

Jacome-Galarza et al. proposed a model for the development and maintenance of osteoclast fusion. Osteoclasts of EMP origin are essential for normal bone development, and their postnatal maintenance is sustained by cell-cell fusion, resulting in the fusion with HSC-derived cells and acquisition of their nuclei, instead of proliferation of the osteoclast precursors (Jacome-Galarza et al., 2019). They showed that mature osteoclasts are long-lived in adults bone depending on interactive fusion between individual HSC-derived circulating cells with locally existing osteoclasts. On the other hand, we found that EMP-derived precursors are long-lived and maintained in extramedullary organs such as in the spleen. In steady state, HSC/EMP-derived osteoclast precursors can fuse in a developing bone. Once an injury happens, unknown factors mobilize EMP-derived osteoclast precursors from the spleen. These precursors travel via blood circulation to participate in fracture repair, by cell-cell fusion between EMP- and local HSC-derived osteoclasts, resulting in multinucleated osteoclasts (Yahara et al., 2020). However, detailed mechanisms of attraction of EMP-derived precursors and cell-cell fusion between EMP- and HSC-derived osteoclast precursors have not yet been elucidated. The life span and maintenance of osteoclast precursors/mature osteoclasts *in vivo* is still under debate.

## Unique Expression Profiles of EMPs- and HSCs-Derived Macrophages and Osteoclast Precursors

EMPs and HSCs provide postnatal macrophages and osteoclast precursors, and they differentiate into mature osteoclasts (Jacome-Galarza et al., 2019; Yahara et al., 2020). However, the functional differences between EMP- and HSC-derived macrophages and osteoclasts are not fully understood. Mass et al. identified the expression of the cytokine receptor RANK [coded by the TNF Receptor Superfamily Member 11a (TNFRSF11A) gene] in pMacs by RNA-seq (Mass et al., 2016). They found that *Rank-Cre; Rosa26eYFP* mice efficiently labeled EMP-derived tissue-resident macrophages but not bone marrow derived-HSCs and their progeny. Interestingly, epithelial cells are also related to the potential epithelial origin of some EMP progenitors (Plein et al., 2018). Because microglia are macrophage-related cells of the central nervous system and originated from embryonic EMPs (Konishi et al., 2020), the majority of postnatal brain microglia expressed eYFP in *Rank-Cre; Rosa26eYFP* mice. It is well-recognized that RANK is the receptor for RANKL and RANK-RANKL signaling essential for osteoclast differentiation and activation. Therefore, osteoclasts and other EMP-derived tissue-resident macrophages such as in brain (microglia), liver (Kupffer cells), and epidermis (Langerhans cells) rise a possibility that they are from same origin. Analysis of the expression profiles of eYFP-negative bone marrow-derived and eYFP-positive EMP-derived macrophages in the postnatal tissue of *Rank-Cre; Rosa26eYFP* mice showed that macrophages from eYFP-positive EMPs expressed high levels of Lyve-1, Stab1, and Gas6, which have been related to homeostatic and anti-inflammatory immune functions. On the other hand, eYFP-negative HSC-derived macrophages characterizes higher expression of Ccr2, which is a marker of HSCs-derived monocyte/macrophage (Weinberger et al., 2020). Use of these markers may be useful in future research to understand the contributions of these populations in homeostasis, tissue repair, and pathology.

## Conclusion and Perspectives

EMP-derived embryonic macrophages persist during adult life and are long-lived cells that can self-renew locally, independent of the HSCs-derived peripheral monocytes. Macrophages derived from the definitive hematopoietic progenitors in the bone marrow are short-lived and are replenished during steady and pathological conditions by monocyte in a CCR2-dependent manner. Monocyte-derived macrophages show distinct gene modifications and profiles compared to the embryonically established macrophages depend on their local tissue environments. However, the principal mechanisms causing the differences in the transcriptomic, epigenomic and their functional signatures between HSCs- and EMPs-derived macrophages and osteoclast remain to be elucidated. Insight into the will inform the optimal functions and distinct roles of EMP-derived osteoclasts in bone homeostasis and repair.

The data reviewed here strongly supports a role for macrophage cells orchestrating fracture repair. Furthermore, heterochronic parabiosis shows that young macrophage cells rejuvenate fracture repair (Vi et al., 2018), and that there is a population of YS derived cells labeled by *Cx3cr1* during embryogenesis that reside in the red pulp of the spleen and are mobilized to bone when injured (Yahara et al., 2020). It is likely that this embryonic population is critical in effective repair, and as this population changes with age, the pace and quality of fracture healing declines. Interestingly, the embryonically derived cell population in the spleen also populates the kidney (Ide et al., 2020), suggesting roles in other processes besides fracture repair. Understanding the role of this embryonic cell population in repair and regeneration will likely have important implications in a variety of reparative and pathologic processes.

## Author Contributions

All authors listed have made a substantial, direct and intellectual contribution to the work, and approved it for publication.

## Conflict of Interest

The authors declare that the research was conducted in the absence of any commercial or financial relationships that could be construed as a potential conflict of interest.
